# Adherence to Mediterranean Diet and its main determinants in a sample of Italian adults: results from the ARIANNA cross-sectional survey

**DOI:** 10.3389/fnut.2024.1346455

**Published:** 2024-02-27

**Authors:** Erica Cardamone, Francesca Iacoponi, Rita Di Benedetto, Giulia Lorenzoni, Annalisa Di Nucci, Federica Zobec, Dario Gregori, Marco Silano

**Affiliations:** ^1^Department of Cardiovascular, Endocrine-metabolic Diseases and Aging, Istituto Superiore di Sanità, Rome, Italy; ^2^Department of Medicine—DMED, Università degli Studi di Udine, Udine, Italy; ^3^Unit of Human Nutrition and Health, Department of Food Safety, Nutrition and Veterinary Public Health, Istituto Superiore di Sanità, Rome, Italy; ^4^Unit of Biostatistics, Epidemiology and Public Health, Department of Cardiac, Thoracic, Vascular Sciences, and Public Health, University of Padova, Padova, Italy; ^5^Zeta Research Ltd., Trieste, Italy

**Keywords:** Adherence to Mediterranean Diet, ARIANNA, MDSS, survey, dietary pattern

## Abstract

**Introduction:**

Over the last years, many Mediterranean countries, including Italy, have witnessed a shift away from the Mediterranean Diet, thus contributing to the high rates of overweight and obesity. The survey “Adherence to Mediterranean Diet in Italy (ARIANNA)” aimed to evaluate the Adherence to Mediterranean Diet (AMD) and its main determinants in the Italian population.

**Materials and methods:**

This study started on March 2023 and was addressed to adults aged ≥17 years, born and resident in Italy, proficient in Italian. Data are collected electronically through a voluntary, anonymous and self-administered questionnaire on the project website. Univariate and then multivariate logistic regressions were performed to evaluate associations between AMD and demographic characteristics, socio-economic status, health status, and lifestyle.

**Results:**

On a total of 3,732 completed questionnaires, the 87.70% of the respondents was female and the 71.28% was 17–40 years old. The 83.82% of the respondents had medium AMD, 11.33% low and only 4.85% high. The multivariate analysis revealed that being male (*p* < 0.001), aged >40 years (*p* < 0.05), workers (*p* ≤ 0.001), and unemployed (*p* < 0.05), determined the probability of having a lower AMD. Vegans and vegetarian’s diets positively contributed to a higher AMD (*p* < 0.001).

**Discussion:**

These results highlighted a medium AMD in the Italian adult participants and suggested the necessity to implement tailored public health intervention strategies to improve food habits.

## 1 Introduction

The Mediterranean Diet (MD) represents one of the healthiest dietary pattern. Its benefits have been recognized by the scientific community since the 1950s with the Seven Countries Study, conducted by the American physiologist Ancel Keys (1–5). MD is a nutritionally adequate plant-centered diet, based on variety and seasonality and characterized by a high intake of vegetables, fruits and nuts, cereals (especially whole-grain cereals), legumes, and olive oil; a moderate-to-high intake of fish; a low-to-moderate intake of dairy products; a low intake of saturated fats, meat and poultry; and a moderate intake of wine (6–8).

The MD, as a role model of a traditional, healthy and sustainable diet, is closely related to the concept of “One Health,” reinforcing the idea that human and ecosystem health are inter-dipendent (7, 9, 10). Indeed, it can essentially bring four benefits to: health, environment, economy, society, and culture (11–13). In terms of health benefits, the adherence to the Mediterranean pattern is able to prevent several chronic non-communicable diseases (NCDs) and the “triple burden” of malnutrition, reduce mortality from all causes, and promote longevity (14, 15). The MD has a low environmental impact, according to use of natural resources (water, soil, and energy) and greenhouse gas emissions, because of the higher intake of plant-based foods (13, 16). In addition, from an economic point of view, it contributes to reducing health expenditure due to the health benefits and prevention of NCDs; enhances the value of the territory and local products by promoting the empowerment of their producers; and helps reduce food waste by being based on frugality (13, 14). In 2010, the United Nations Educational, Scientific and Cultural Organization (UNESCO) acknowledged the MD as an Intangible Cultural Heritage of Humanity for its principles of conviviality, daily rituals, social and gastronomic practices, and celebrations (5, 17). In the Mediterranean model, food is a tool for social relations, regardless of age and class, and thanks to its link to the land, seasonality and biodiversity, it allows for greater awareness and responsibility for food (5).

Despite the well-documented advantages of the MD, in the past 10 years, many countries in the Mediterranean area, including Italy, are experiencing a progressive shift away from this dietary model, along with a process of “westernization” of food habits (7, 18–21). This phenomenon has contributed to the high rates of overweight and obesity found in national surveillance systems of all age groups in the Italian population (22). In particular, in Italy, the percentage of overweight and obesity is 29.7% in children (23), 22.6% in adolescents (24), and 42.90% in adults (25).

Over the years, numerous indexes and scores have been developed to evaluate the Adherence to Mediterranean Diet (AMD), starting with the first and most widely used score proposed by Trichopoulou et al. (26–28). These scoring systems, based on the combination of food components included, represent useful tools in the study of diet and its health benefits (27–29).

Recent surveys of the Italian population, using different scores and methodologies, have revealed a picture ranging from low (30–33) to moderate (7, 19) AMD. Determinants of this dietary pattern often found in Italy include high cultural level and age, with elderly associated with greater AMD (34). Confirming this, studies on the Italian youth population show low rates of good AMD (35, 36), although there is recent evidence that has found no association between AMD and age (7), and no difference in AMD according to the age (18).

Clarity is needed to understand the socio-demographic factors and more generally the determinants associated with AMD in Italy. Among the various factors to be studied, there is also adherence to particular and restrictive diets (food allergies, coeliac disease, vegetarianism, and veganism). Indeed, collecting this data would mean being able to assess the influence of these types of diets on AMD and their impact on dietary adequacy in terms of foods and patterns. To the best of our knowledge, looking at the adherence to different special diets in relation to AMD has not been done before in surveys targeting the entire Italian population.

It seems important to investigate the current state of AMD in Italy and to provide the national policy makers the findings, to implement evidence-based interventions, and to fulfill the commitments of the UN Decade of Action on Nutrition 2016–2025 (37) and the Sustainable Development Goals of the UN 2030 Agenda (38).

Based on these considerations, the Adherence to the Mediterranean Diet in Italy (ARIANNA) survey aimed to evaluate the AMD and its main socio-demographic and health-related determinants in the Italian population, using a score system validated and described in scientific literature, and to report the national policy makers the results.

## 2 Materials and methods

### 2.1 Study design

The ARIANNA project has been approved by Ethics Committee of the Istituto Superiore di Sanità (approval n AOO 0028080 on 27 July 2021). A detailed study protocol was defined, in which sample size, recruitment methods, and AMD assessment were carefully explained (6).

### 2.2 Sample and data collection

A cross-sectional survey was carried out on voluntary respondents recruited between March and April 2023 from the Italian population.

According to the ARIANNA survey inclusion criteria, participants in this study had to be people aged ≥17 years, born and resident in Italy, and proficient in Italian.

Data were collected electronically through a validated questionnaire on the project website (39). The online software used has been developed by Zeta Research Ltd. (Trieste, Italy). Involvement of the participants was boosted through media, social network and press-campaign. Participation in the study was fully voluntary, anonymous and free. All subjects were required to give their consent in advance for the collection and processing of personal data in accordance with the European Commission General Data Protection Regulation (679/2016), before accessing the questionnaire.

The self-administered questionnaire in the ARIANNA project consists of two different sections. The initial part includes questions on demographic factors, socio-economic status, health status, and lifestyle. The second one includes questions on participants’ dietary habits (6). The response formats of the first section are closed and structured for: sex, age, qualification, adherence to special diets, main concomitant pathology, annual income, and occupation. The occupation item is completed with two more questions on the type of employment contract. Geographical areas of birth and residence, number of household members, and frequency of physical activity are also requested. All answers in the questionnaire are mandatory, except for annual income, which is an optional item (6). In the second section, frequency of intake of 14 food groups is asked participants as usual number of times of consumption per day (fruit, vegetables, cereals, olive oil, and dairy products) and per week (dried fruit and nuts, potatoes, legumes, eggs, fish, white meat, red meat, sweets, wine, or beer) (6), over the past year.

The ARIANNA questionnaire in English language is reported as Supplementary material.

### 2.3 Adherence to Mediterranean Diet

To assess the AMD of participants aged ≥17 years, the *a priori* score system Mediterranean Diet Serving Score (MDSS) was chosen.

The MDSS is an easy, valid, and accurate tool proposed by Monteagudo et al. (40). This was one of the few AMD score systems that has been compared with the one created by Trichopoulou et al. (26), showing high levels of agreement (27, 32). It consists of 14 items, and it is based on consumption frequency of food. Intakes that fall within the number of recommended servings are awarded a score of 3, 2, or 1 point for recommendations expressed in times/meal, times/day, or times/week, respectively. A score of 0 is given when the intake is higher or lower than the recommendation. In adults, 1 point is added for alcohol consumption equivalent to 1 and 2 glasses of fermented beverages for females and males, respectively (see Table 1). Indeed, the MDSS total score ranges between 0 and 24 points for adults/elderly, and between 0 and 23 for adolescents (40).

**Table 1:** Mediterranean Diet Serving Score items (40).

	Recommendation^*^	Score
Fruit	1–2 servings/main meal^**^	3
Vegetables	≥ 2 servings/main meal^**^	3
Cereals^a^	1–2 servings/main meal^**^	3
Potatoes	≤ 3 servings/week	1
Olive oil^b^	1 serving/main meal^**^	3
Nuts	1–2 servings/day	2
Dairy products^c^	2 servings/day	2
Legumes	≥ 2 servings/week	1
Eggs	2–4 servings/week	1
Fish	≥ 2 servings/week	1
White meat^d^	2 servings/week	1
Red meat^e^	< 2 servings/week	1
Sweets^f^	≤ 2 servings/week	1
Fermented beverages^g^	1–2 glass/day	1
Total score		24

^*^According to the Mediterranean Diet Pyramid (41). ^**^Main meals: breakfast, lunch, and dinner. ^a^Bread, breakfast cereals, rice, and pasta; ^b^Olive oil used on salads or bread or for frying; ^c^Milk, yoghurt, cheese, and ice-cream; ^d^Poultry; ^e^Pork, beef, or lamb; ^f^Sugar, candies, pastries, sweetened fruit juices, and soft drinks; and ^g^Wine and beer.

The final score was obtained by summing each individual score and the range between 0 and 24 was considered for all questionnaire respondents aged ≥17 years. In addition, according to the obtained level of AMD (tertiles), the respondents were divided into low (first tertile, MDSS score ≤ 5), medium (second tertile, MDSS score 6–10), and high (third tertile, MDSS score ≥ 11) adherence groups, to better distinguish between adherents and non-adherents, as previously described by Monteagudo et al. (32, 40).

Once a participant had completed the questionnaire, his/her score was shown on the screen. For those that obtained an AMD score low or medium, some useful information and suggestions to increase their adherence were provided through links to the project website. Respondents with high AMD, on the other hand, were invited to continue following healthy eating habits (6).

### 2.4 Statistical analysis

Based on the data collected, all variables were assessed as categorical variables and were re-categorized: age groups (17–40 years, >40 years), education (≤13 years, >13 years), annual income (<15.000€, 15.000–30.000€, 30.000–50.000€, and >50.000€), occupation (non-workers/students, unemployed, part-time workers, and full-time workers), family size (1 person, 2 people, 3 people, 4 people, and 5 or more people), health conditions (none, cardiometabolic diseases, chronic obstructive pulmonary disease, and cancer), adherence to special diet (none, celiac disease, food allergy, lactose intolerance, vegans/vegetarians, and religious reasons), frequency of physical activity (<2.5 h/week, ≥2.5h/week). Geographical areas of birth and residence were reported according to the Nomenclature of Territorial Units for Statistics (NUTS 1) (42).

A comparison by *χ*^2^ test related the distribution of participant’s sex, age and geographical residence was made with the database of the Italian National Institute of Statistics (ISTAT) to verify the likelihood of the data collected at national level (43).

Data were reported as absolute frequencies and percentages (%). Comparison between geographic area of residence regarding to socio-demographic and health characteristics were performed using *χ*^2^ test or Fisher’s exact test. Percentages were reported without counting of missing data.

Furthermore, *χ*^2^ test was also used to assess the association between variables characterizing the socio-economic status such as education, annual income, occupation, and family size.

The associations between the outcome AMD and the variables of interest were evaluated by *χ*^2^ test followed by an ordered logistic regression (OLR) analysis. At first, univariate OLR analyses were performed. The variables statistically associated with the level of AMD (high vs. medium/low and high/medium vs. low) were then included into a multivariate OLR model, using the backward stepwise method and setting the inclusion level to 0.2 (44). To assess the degree of associations, odds ratios (OR) and 95% confidence intervals (CI) were estimated.

The level of significance was established at *p* < 0.05. All the analyses were performed using the statistical software StataSE V.17 for Windows (StataCorp) and Microsoft Excel 2016 was used to create chart.

## 3 Results

A total of 3,732 voluntary adult participants took part in the online survey. The 87.70% (3,273) were females and 12.30% (459) males. The 71.28% was from 17 to 40 years old, the rest of the respondents (28.72%) was over 40 years old. The only variable with missing data is the optional annual income item with 22.75% missing values.

The sample description according to geographical areas of residence is shown in Table 2. More than half of the subjects were resident in northern Italy (36.60% in the Northwest and 18.78% in the Northeast), 26.21% lived in the Center, 12.08% in the South, and the remaining 6.32% in the Islands.

**Table 2:** Demographic characteristics, socio-economic status, health status, and lifestyle, according to geographical areas of residence.

Sample characteristics	Northwest	Northeast	Center	South	Islands	*p* value^*^
	*n* = 1,366	*n* = 701	*n* = 978	*n* = 451	*n* = 236	
Sex						0.060
Female	1,174 (85.94)	620 (88.45)	867 (88.65)	395 (87.58)	217 (91.95)	
Male	192 (14.06)	81 (11.55)	111 (11.35)	56 (12.42)	19 (8.05)	
Age groups						< 0.001
17–40 years	1,001 (73.28)	540 (77.03)	560 (57.26)	368 (81.60)	191 (80.93)	
>40 years	365 (26.72)	161 (22.97)	418 (42.74)	83 (18.40)	45 (19.07)	
Education						< 0.001
≤13 years	582 (42.61)	316 (45.08)	307 (31.39)	187 (41.46)	104 (44.07)	
>13 years	784 (57.39)	385 (54.92)	671 (68.61)	264 (58.54)	132 (55.93)	
Annual income						< 0.001
<EUR 15.000	229 (21.64)	179 (34.03)	181 (23.36)	139 (41.87)	85 (44.97)	
EUR 15.000–30.000	411 (38.85)	221 (42.02)	282 (36.25)	117 (35.24)	68 (35.98)	
EUR 30.000–50.000	277 (26.18)	88 (16.73)	251 (32.26)	57 (17.17)	23 (12.17)	
>EUR 50.000	141 (13.33)	38 (7.22)	64 (8.23)	19 (5.72)	13 (6.88)	
*missing*	308	175	200	119	47	
Occupation						< 0.001
Non-workers/Students	255 (18.67)	194 (27.67)	202 (20.65)	162 (35.92)	79 (33.47)	
Unemployed	42 (3.07)	24 (3.42)	43 (4.40)	37 (8.20)	27 (11.44)	
Part-time workers	193 (14.13)	115 (16.41)	121 (12.37)	72 (15.96)	36 (15.25)	
Full-time workers	876 (64.13)	368 (52.50)	612 (62.58)	180 (39.91)	94 (39.83)	
Family size						< 0.001
1 person	220 (16.11)	107 (15.26)	119 (12.17)	37 (8.20)	23 (9.75)	
2 people	385 (28.18)	161 (22.97)	257 (26.28)	82 (18.18)	49 (20.76)	
3 people	308 (22.55)	161 (22.97)	257 (26.28)	104 (23.06)	54 (22.88)	
4 people	333 (24.38)	190 (27.10)	251 (25.66)	147 (32.59)	67 (28.39)	
5 or more people	120 (8.78)	82 (11.70)	94 (9.61)	81 (17.96)	43 (18.22)	
Health conditions						0.109
None	1,291 (94.51)	669 (95.44)	901 (92.13)	426 (94.46)	229 (97.03)	
Cardiometabolic diseases	56 (4.10)	21 (3.00)	56 (5.73)	21 (4.66)	6 (2.54)	
Chronic obstructive pulmonary disease	3 (0.22)	1 (0.14)	5 (0.51)	0.00	0.00	
Cancer	16 (1.17)	10 (1.43)	16 (1.64)	4 (0.89)	1 (0.42)	
Adherence to special diet						0.563
None	1,134 (83.02)	573 (81.74)	814 (83.23)	370 (82.04)	194 (82.20)	
Celiac disease	34 (2.49)	16 (2.28)	18 (1.84)	7 (1.55)	7 (2.97)	
Food allergy	33 (2.42)	7 (1.00)	18 (1.84)	10 (2.22)	5 (2.12)	
Lactose intolerance	97 (7.10)	59 (8.42)	83 (8.49)	45 (9.98)	21 (8.90)	
Vegans/vegetarians	64 (4.69)	44 (6.28)	43 (4.40)	19 (4.21)	8 (3.39)	
Religion reasons	4 (0.29)	2 (0.29)	2 (0.20)	0.00	1 (0.42)	
Physical activity						0.183
< 2.5 h/week	635 (46.49)	305 (43.51)	476 (48.67)	225 (49.89)	109 (46.19)	
≥ 2.5 h/week	731 (53.51)	396 (56.49)	502 (51.33)	226 (50.11)	127 (53.81)	

Data are expressed as absolute frequencies and percentages (%), calculated on the total of subjects excluding missing data. ^*^Chi-square test.

The demographic characteristics of the sample, such as sex, age, and geographical area of residence, were statistically different (*p* < 0.001) from those of the distribution of the Italian population reported by ISTAT (43).

No significance was observed among geographical areas of residence for sex (*p* = 0.060), health conditions (*p* = 0.109), adherence to special diets (*p* = 0.563), and frequency of physical activity (*p* = 0.183).

Central Italy, compared to the other macro-areas, was characterized by a greater percentage of respondents aged over 40 years (42.74%; *p* < 0.001) and a higher prevalence of subjects with an educational level beyond 13 years of study (68.61%; *p* < 0.001). In the South and Islands, younger respondents prevailed by a large margin (81.60 and 80.93%, respectively). About annual income and occupation, there were significant differences across the country (*p* < 0.001). Specifically, a higher prevalence of subjects with an annual income >30.000€ and of full-time workers was observed in the Northwest and Center, while in the South and Island a lower annual income and job stability were observed. Compared to the rest of the country, Southern Italy and Island were also characterized by a higher prevalence of large families with four members or more.

The levels of AMD according to the sample characteristics are reported in Table 3. The 83.82% of the respondents had medium AMD, 11.33% had low AMD, and only 4.85% had high AMD.

**Table 3:** Levels of Adherence to Mediterranean Diet (AMD), according to the Mediterranean Diet Serving Score (MDSS), stratified by main sample characteristics.

	AMD				
Sample characteristics	Low	Medium	High	p value^*^	All
	*n* = 423	*n* = 3,128	*n* = 181		*n* = 3,732
Sex				< 0.001	
Female	314 (9.59)	2,789 (85.21)	170 (5.19)		3,273 (87.70)
Male	109 (23.75)	339 (73.86)	11 (2.40)		459 (12.30)
Age groups				< 0.001	
17–40 years	252 (9.47)	2,270 (85.34)	138 (5.19)		2,660 (71.28)
> 40 years	171 (15.95)	858 (80.04)	43 (4.01)		1,072 (28.72)
Education				0.006	
≤ 13 years	196 (13.10)	1,239 (82.82)	61 (4.08)		1,496 (40.09)
> 13 years	227 (10.15)	1,889 (84.48)	120 (5.37)		2,236 (59.91)
Annual income				0.111	
<EUR 15.000	93 (11.44)	685 (84.26)	35 (4.31)		813 (28.20)
EUR 15.000–30.000	128 (11.65)	914 (83.17)	57 (5.19)		1,099 (38.12)
EUR 30.000–50.000	92 (13.22)	578 (83.05)	26 (3.74)		696 (24.14)
>EUR 50.000	48 (17.45)	217 (78.91)	10 (3.64)		275 (9.54)
*missing*	62	734	53		849
Occupation				< 0.001	
Non-workers/Students	47 (5.27)	789 (88.45)	56 (6.28)		892 (23.90)
Unemployed	18 (10.40)	147 (84.97)	8 (4.62)		173 (4.64)
Part-time workers	57 (10.61)	459 (85.47)	21 (3.91)		537 (14.39)
Full-time workers	301 (14.13)	1,733 (81.36)	96 (4.51)		2,130 (57.07)
Family size				0.163	
1 person	48 (9.49)	427 (84.39)	31 (6.13)		506 (13.56)
2 people	113 (12.10)	778 (83.30)	43 (4.60)		934 (25.03)
3 people	106 (11.99)	749 (84.73)	29 (3.28)		884 (23.69)
4 people	116 (11.74)	817 (82.69)	55 (5.57)		988 (26.47)
5 or more people	40 (9.52)	357 (85.00)	23 (5.48)		420 (11.25)
Macro-area of birth				0.213	
Northwest	169 (13.05)	1,061 (81.93)	65 (5.02)		1,295 (34.70)
Northeast	77 (11.31)	563 (82.67)	41 (6.02)		681 (18.25)
Center	95 (10.36)	784 (85.50)	38 (4.14)		917 (24.57)
South	57 (10.04)	487 (85.74)	24 (4.23)		568 (15.22)
Islands	25 (9.23)	233 (85.98)	13 (4.80)		271 (7.26)
Macro-area of residence				0.117	
Northwest	178 (13.03)	1,119 (81.92)	69 (5.05)		1,366 (36.60)
Northeast	80 (11.41)	580 (82.74)	41 (5.85)		701 (18.78)
Center	105 (10.74)	830 (84.87)	43 (4.40)		978 (26.21)
South	41 (9.09)	393 (87.14)	17 (3.77)		451 (12.08)
Islands	19 (8.05)	206 (87.29)	11 (4.66)		236 (6.32)
Health conditions				0.227	
None	388 (11.04)	2,955 (84.04)	173 (4.92)		3,516 (94.21)
Cardiometabolic diseases	25 (15.63)	130 (81.25)	5 (3.13)		160 (4.29)
Chronic obstructive pulmonary disease	1 (11.11)	8 (88.89)	0.00		9 (0.24)
Cancer	9 (19.15)	35 (74.47)	3 (6.38)		47 (1.26)
Adherence to special diet				< 0.001	
None	379 (12.29)	2,568 (83.24)	138 (4.47)		3,085 (82.66)
Celiac disease	7 (8.54)	71 (86.59)	4 (4.88)		82 (2.20)
Food allergy	8 (10.96)	64 (87.67)	1 (1.37)		73 (1.96)
Lactose intolerance	27 (8.85)	267 (87.54)	11 (3.61)		305 (8.17)
Vegans/vegetarians	0.00	151 (84.83)	27 (15.17)		178 (4.77)
Religion reasons	2 (22.22)	7 (77.78)	0.00		9 (0.24)

Data are expressed as absolute frequencies and percentages (%), calculated on the total of subjects excluding missing data. MDSS score: low ≤ 5; medium 6–10; high ≥ 11. ^*^Chi-square test.

There was no significant association between AMD and annual income (*p* = 0.111), family size (*p* = 0.163), macro-area of birth (*p* = 0.213) and residence (*p* = 0.117), and health conditions (*p* = 0.227). The prevalence rate of low adherence was significantly higher in males (23.75%; *p* < 0.001), in respondents aged >40 years (15.95%; *p* < 0.001), in those with a lower level of education (13.10%; *p* < 0.01), and in full-time workers (14.13%; *p* < 0.001). Furthermore, a higher prevalence of high AMD was observed among vegans and vegetarians (15.17%; *p* < 0.001), and none of them had low adherence.

In the univariate OLR analysis sex, age groups, education, occupation, and adherence to special diet were statistically associated with the AMD (high vs. medium/low and high/medium vs. low), as shown in Table 4. Being male (*p* < 0.001), aged >40 years (*p* < 0.05), workers (*p* ≤ 0.001), and unemployed (*p* < 0.05) increased the probability of having a lower AMD; while being vegans and vegetarians (*p* < 0.001) positively determined the AMD, as it was found in the multivariate OLR model. The other categories of adherence to special diet variable were not significant (Table 4).

**Table 4:** Ordered logistic regression analysis for being in the high vs. medium/low and high/medium vs. low categories of Adherence to Mediterranean Diet (AMD), by considering the variables alone (univariate analysis) or together (multivariate analysis).

	Univariate analysis		Multivariate analysis	
Variables	OR (95% CI)	*p* value	OR (95% CI)	*p* value
Sex				
Female	*Ref.*		*Ref.*	
Male	0.351 (0.277–0.444)	< 0.001	0.418 (0.325–0.536)	< 0.001
Age groups				
17–40 years	*Ref.*		*Ref.*	
> 40 years	0.595 (0.492–0.719)	< 0.001	0.807 (0.660–0.986)	0.036
Education				
≤ 13 years	*Ref.*		-	-
> 13 years	1.334 (1.118–1.593)	0.001	-	-
Occupation				
Non-workers/Students	*Ref.*		*Ref.*	
Unemployed	0.607 (0.386–0.954)	0.031	0.614 (0.390–0.969)	0.036
Part-time workers	0.570 (0.422–0.768)	< 0.001	0.596 (0.440–0.808)	< 0.01
Full-time workers	0.463 (0.370–0.580)	< 0.001	0.580 (0.459–0.733)	< 0.001
Adherence to special diet				
None	*Ref.*		*Ref.*	
Celiac disease	1.353 (0.730–2.508)	0.337	1.188 (0.639–2.211)	0.585
Food allergy	0.902 (0.489–1.663)	0.741	0.848 (0.456–1.578)	0.603
Lactose intolerance	1.208 (0.872–1.674)	0.255	1.095 (0.787–1.524)	0.589
Vegans/vegetarians	4.927 (3.338–7.273)	< 0.001	4.266 (2.876–6.326)	< 0.001
Religious reasons	0.438 (0.099–1.933)	0.276	0.336 (0.075–1.512)	0.155

OR, Odds ratio; CI, Confidence interval; Ref., Reference category.

A significant association was found among socio-economic status variables (*p* < 0.001): subjects with a higher educational level had a higher job stability, a higher annual income, and smaller families. For this reason, the education variable was not included in the final multivariate OLR model.

**Figure 1:**
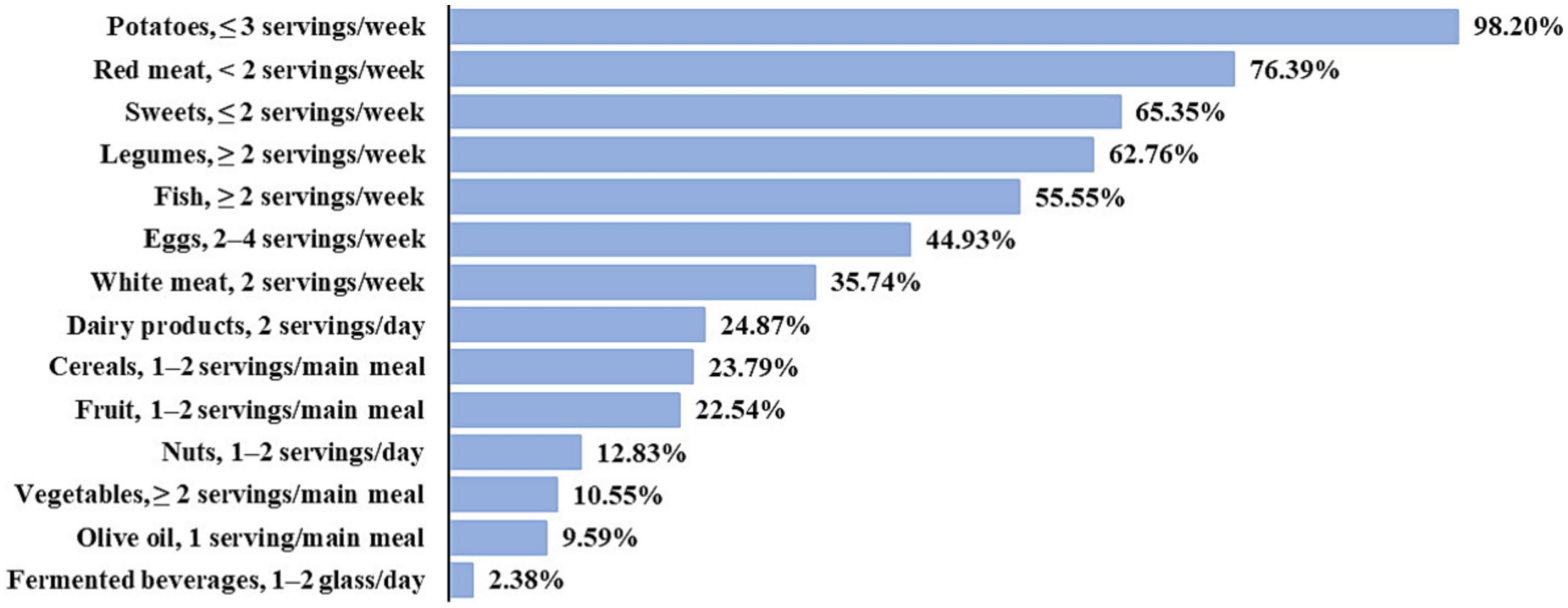
Answers in line with Adherence to Mediterranean Diet (AMD), according to MDSS. The remaining part of respondents consumed food more or less frequently than recommended.

Figure 1 shows the percentages of answers according to the recommended food consumption frequencies on which the MDSS is based. Most of the sample followed the recommendation for potatoes and red meat (98.20% and 76.39%, respectively). The 65.35% of the subjects complied with the recommended frequency of consumption for sweets. Legumes and fish were consumed according to the recommendation by the 62.76% and the 55.55% of the sample, respectively. About other typically Mediterranean foods, only the 23.79% complied with the recommendation for cereals, the 22.54% for fruit, 12.83% for nuts, 10.55% for vegetables, and 9.59% for olive oil. Just over one third of the sample (35.74%) consumed white meat at a frequency of 2 servings/week. Recommendation for eggs was followed by 44.93% of the subjects, and for dairy products by 24.87%. Only 2.38% of respondents reported an intake of fermented beverages of 1–2 glass/day.

## 4 Discussion

The ARIANNA study investigated the AMD in a sample of adult subjects residing in Italy. To the best of our knowledge, this is the first updated nation-wide study that attempted to evaluate AMD and its associated factors in a large number of participants.

In general, a medium AMD was found, in line with some recent surveys on the Italian adult population even though the assessment was carried out using different tools and methodologies (7, 19). These results would seem to be in contrast with other recent studies that have reported a low AMD in Italy (30–33). However, only 4.85% of the ARIANNA study sample showed a high AMD, indicating a general shift away from the Mediterranean dietary pattern of the Italian population. Indeed, this is supported by a recent systematic literature review conducted with the aim of providing an overview of AMD among populations of Mediterranean countries that pointed out a low to moderate AMD in the past 10 years (18). Specifically, looking at individual countries, data from a national survey in Greece also confirm that the adult Greek population has headed away from the traditional MD (45). On the other hand, although two recent prospective analyses have shown an improvement in AMD over a 10-year period in both Spain and France, the average AMD shown is moderate, in line with the results of this study (46, 47).

Analysis of the data obtained from this national research showed that female sex, young age, studying and not working, vegetarianism, and veganism were significantly associated with greater AMD.

Similar to what was found by other recent Italian surveys, AMD was better in females than in males (7, 19, 30). This aspect, as well as the higher percentage of female respondents, could be explained by the fact that women seem to be more interested and attentive to nutrition and have more nutritional knowledge (30, 48–50).

The present study showed that participants aged >40 years had lower AMD, in contrast to the findings of several studies in which a significative association between adults/elderly and higher adherence has been reported (19, 30, 33, 34, 51–53). The results of ARIANNA study joined the work of Foscolou et al. (54) that revealed a reduction in AMD among the older population over the years correlated to several social determinants (18). Another determinant of adherence to the Mediterranean dietary pattern found is a higher level of education, in agreement with other similar Italian investigations (7, 19, 27, 30, 45, 46, 48). Furthermore, the present research revealed a negative association between part-time and full-time work and a higher AMD, compared to students and non-workers. In line with these findings, Marventano et al. (55) found that high adherence was directly associated with education and inversely with high occupational status. Hypothesis developed to motivate the results of the ARIANNA study is that working subjects probably lose the advantage given by their educational level and tend to have a lower AMD, due to the longer time spent outside home and consequently less time to devote to cooking and healthy eating habits.

Comparing these results with others from different Mediterranean countries, Obeid et al. (18) showed a shift away from the MD in recent years and observed no clear differences between sex and age groups. The authors, in fact, concluded by pointing out that further research is needed on the AMD scores across ages and determinants for shifting away from the MD (18).

In Italian surveys similar to this one, adherence to various special diets has not been explored in relation to the Mediterranean dietary model before, opening the way to further investigations. Participants following a vegan and vegetarian diet showed higher levels of AMD than other evaluated categories, considered as omnivores. This, as shown by Avital et al. in a recent study carried out on an Israeli population sample, is probably due to the tendency of omnivores to consumption of more animal-based foods (meat, eggs, and dairy products), the higher intake of plant-based products by vegans and vegetarians, and differences in nutrition knowledge between omnivores and vegetarians (56). Another aspect that we would like to highlight concerns the AMD of celiac respondents. In fact, celiac disease (CD) does not seem to be significantly associated with higher AMD. In support of this, a recent study found a low AMD score and an average adherence score even lower than that of healthy participants in a group of Italian adults with CD enrolled in Northern Italy, probably related to a high intake of gluten-free processed foods with a low nutritional quality and general unhealthy food choices to balance the withdrawal of gluten-containing foods from the diet (57, 58). Finally, a significant association with AMD was also not found for food allergies and lactose intolerance. It is likely that these conditions significantly influence the frequencies of consumption of specific individual foods, without altering adherence to the dietary pattern as a whole.

The AMD was not significantly associated with geographical area of residence and birth, and a certain homogeneity was observed throughout the country, like to the findings of Biasini et al. (7). However, other recent investigations in Italy showed a significant association with the geographical location (30, 33). In contrast to what was found by Aureli et al. (30) also no association was observed with family composition. Several studies pointed out income as a factor influencing AMD, but in this national survey no association was found (7, 30, 52, 59). We recorded a 22.75% of non-respondent subjects who refused to declare or did not know their annual income or preferred not to answer, as it was an optional item. Given the sensitivity of the issue, having a high number of nonresponses is quite common in this type of survey (59, 60).

An initial descriptive analysis of the answers related to the consumption frequencies of single food groups shows a low percentage of the sample complied with the MDSS-based recommendations for cereals (23.79%), fruit (22.54%), nuts (12.83%), and vegetables (10.55%), in agreement with Aureli et al. (30). Also, the recommendation for olive oil was followed only by 9.59% of the sample, as reported by Biasini et al. (7) and Veronese et al. (61). Furthermore, analogous to the results of Barnaba et al. (32), the 98.20% of the sample followed the recommendation for red meat and the lowest percentage of respondents was reported for fermented beverages (2.38%). However, further investigations are needed, comparing the answers from the questionnaire with the current recommendations in Italy (62).

The present findings suggest the necessity to implement tailored public health policies and interventions to promote the MD. In particular, it would be useful to launch promotion campaigns on healthy lifestyles and dietary habits and to introduce nudging policies in specific settings, such as workplaces and universities. These actions could help to increase health literacy, in particular food and nutrition literacy, to which the World Health Organization (WHO) has recognized a central role in preventing and controlling NCDs (63).

This analysis has some limitations that should be discussed. A major limit is that the generalizability of the present findings should be made with caution. Indeed, this is confirmed by the difference in sex, age and geographical area of residence between the distribution of the sample and that of the Italian population. The survey participants were not selected, completed the questionnaire on their own initiative, and their recruitment was boosted through media and press campaign, leading to a gender imbalance as a potential bias. In addition, participation was only aimed at those who had access to Internet and electronic devices (smartphone, computer, and tablet). The use of a self-administered questionnaire may have led to recall and misclassification bias. Another limitation is represented by the cross-sectional nature of the study. Finally, the tool used to collect food data and assess AMD (40) was brief, easy and rapid and, due to its brevity, may not have captured the complexity of the dietary pattern. Indeed, indexes are usually based on data acquired within more precise and comprehensive tools (e.g., 24 h quantitative intake recall, dietary records, and food frequency questionnaires) (64), and Monteagudo et al. themselves (40) constructed the MDSS on a broader food frequency questionnaire. Actually, this tool was later validated in a Croatian sample and compared with another brief questionnaire that was shown to be valid for assessing AMD (65, 66), concluding that MDSS questionnaire could be used as a screening tool in the general population for public health surveillance, in clinical settings, and in scientific studies (64). Moreover, MDSS (40) does not include questions about food items servings and this may have biased the responses from the participants.

Despite these limitations, the ARIANNA study has some strengths, including the large sample size and the use of an accurate and easy web-based questionnaire designed to measure AMD. It is a nation-wide study that collected information not only on demographic factors, socio-economic status, health status, and lifestyle, but also on adherence to special diets. Furthermore, to the best of our knowledge, this is the first survey-based study carried out in Italy specifically designed to provide policy makers the results to implement evidence-based interventions.

In summary, the present study appraised the AMD in a sample of adult individuals (≥17 years) born and resident in Italy. Overall, this research found a medium AMD in the target population. The study of associations with socio-demographic and health factors revealed that female sex, young age (17–40 years old), studying and not working, vegan and vegetarian diets were likely determinants of a higher AMD. On the contrary males, subjects aged >40 years, workers and unemployed need to improve their food habits to come closer to the Mediterranean dietary pattern.

The present findings emphasize the importance of reporting the current picture to national policy makers and suggest the necessity to implement tailored intervention strategies. These actions should aim to undertake evidence-based communication and education interventions at national level and promote healthy eating habits, to get closer to traditional dietary patterns and in particular to MD.

## Data availability statement

The original contributions presented in the study are included in the article/supplementary material, further inquiries can be directed to the first/corresponding authors.

## Ethics statement

The study involving humans was approved by Ethics Committee of the Istituto Superiore di Sanità (approval n AOO 0028080 on 27 July 2021). The study was conducted in accordance with the local legislation and institutional requirements. Written informed consent for participation in this study was provided by the participants and their legal guardians/next of kin.

## Author contributions

EC: Data curation, Methodology, Validation, Writing – original draft. FI: Data curation, Formal analysis, Methodology, Validation, Writing – original draft. RDB: Methodology, Writing – review & editing. GL: Data curation, Methodology, Writing – review & editing. ADN: Validation, Writing – review & editing. FZ: Software, Writing – review & editing. DG: Conceptualization, Formal analysis, Project administration, Writing – review & editing. MS: Conceptualization, Funding acquisition, Project administration, Supervision, Writing – original draft.
