# Supplementary material for: Adherence to Mediterranean Diet and its main determinants in a sample of Italian adults: results from the ARIANNA cross-sectional survey
Source: Front Nutr. 2024 Feb 27;11:1346455. doi: 10.3389/fnut.2024.1346455 (PMC10927747; doi:10.3389/fnut.2024.1346455)
Supplement: Supplementary file 1 [file Data_Sheet_1.PDF]

Supplementary Material

ARIANNA questionnaire

Sex:

- Male
- Female

Age:

- ≤16 years
- 17-20 years
- 21-40 years
- 41-60 years
- 61-80 years
- ≥81 years

Geographical area of birth:

- Province
- Zip code

Geographical area of residence:

- Zip code

Number of members within the household:

- Number of adults (>18 years)
- Number of youth (13-18 years)
- Number of children (≤12 years)

Adherence to special diets:

- None
- Lactose intolerance
- Celiac disease
- Vegetarianism
- Veganism
- Food allergy (specify which)
- Religious reasons (specify which)

Concomitant pathology (main):

- None listed
- Type 2 diabetes
- Hypertension
- Myocardial infarction
- Stroke
- Chronic obstructive pulmonary disease
- Cancer

Physical activity:

- Number of hours per week

Qualification:

- None
- Primary school certificate
- Lower secondary school certificate
- High school diploma
- Degree
- Postgraduate study

Annual income (optional):

- < 5.000€
- 5.000-15.000€
- 15.000-30.000€
- 30.000€-50.000€
- 50.000€-100.000€
- >100.000

Occupation:

- None (non-worker or student, excluding unemployed)
- Employment
- Coordinated and continuous collaboration (with or without project)
- Occasional work
- Self-employment (self-employed, entrepreneur, freelancer)
- Unemployment (unemployed or on lay-off)

If employed:

- Fixed-term
- Permanent

It is a job:

- Full-time (>36h/wk)
- Part-time (<36h/wk)

KIDMED (≤16 years):

- Takes a fruit or fruit juice every day
- Has a second fruit every day
- Has fresh or cooked vegetables regularly once a day
- Has fresh or cooked vegetables more than once a day
- Consumes fish regularly (at least 2–3/week)
- Goes >1/ week to a fast food restaurant (hamburger)
- Likes pulses and eats them >1/week
- Consumes pasta or rice almost every day (5 or more per week)
- Has cereals or grains (bread, etc) for breakfast
- Consumes nuts regularly (at least 2–3/week)
- Uses olive oil at home
- Skips breakfast
- Has a dairy product for breakfast (yoghurt, milk, etc)
- Has commercially baked goods or pastries for breakfast
- Takes two yoghurts and/or some cheese (40 g) daily
- Takes sweets and candy several times every day

MDSS (≥17 years):

- Fruits (numbers of times per day)
- Vegetables (numbers of times per day)
- Cereals (numbers of times per day)
- Potatoes (numbers of times per week)
- Olive oil (numbers of times per day)
- Nuts (numbers of times per week)
- Dairy products (numbers of times per day)
- Legumes (numbers of times per week)
- Eggs (numbers of times per week)
- Fish (numbers of times per week)
- White meat (numbers of times per week)
- Red meat (numbers of times per week)
- Sweets (numbers of times per week)
- Fermented beverages (numbers of times per week)
